# Assessing the risk of malaria re-transmission in Anhui Province, China: an integration of spatiotemporal scan statistics and ecological niche modeling

**DOI:** 10.1186/s12879-026-12783-z

**Published:** 2026-02-06

**Authors:** Bowen Liu, Tao Zhang, Jingbo Xue, Shiyi Huo, Houliang He, Xiao Tan, Weidong Li, Shizhu Li

**Affiliations:** 1 School of Global Health, Chinese Centre for Tropical Diseases Research, Shanghai Jiao Tong University School of Medicine, Shanghai, 200025 China; 2 Yangpu District Center for Disease Control and Prevention (Yangpu District Health Supervision Institute), Shanghai, 200090 China; 3 Anhui Provincial Center for Disease Control and Prevention, 12560 Fanhua Road, Anhui, Hefei, 230601 China; 4 National Institute of Parasitic Diseases, Chinese Center for Disease Control and Prevention, Chinese Center for Tropical Diseases Research, National Key Laboratory of Intelligent Tracking and Forecasting for Infectious Diseases, Key Laboratory on Parasite and Vector Biology, National Health Commission, WHO Collaborating Centre for Tropical Diseases, National Center for International Research on Tropical Diseases, Ministry of Science and Technology, Shanghai, 200025 China

**Keywords:** Imported malaria, Biomod2, Spatial epidemiology, Prevention of re-transmission of malaria, Anhui Province, China

## Abstract

**Objective:**

This study aims to identify hotspots of imported malaria cases and areas suitable for malaria vector distribution in Anhui Province. By visualizing the province-wide risk of malaria re-transmission, the research provides a scientific basis for implementing stratified and targeted strategies to prevent malaria reintroduction.

**Methods:**

Epidemiological data on imported malaria cases and demographic information from Anhui Province, spanning 2017 to 2023, were collected alongside vector surveillance data from 2023. ArcGIS10.8 and SaTScan10.1.2 were used to analyze the spatial-temporal characteristics of the imported cases, and R4.4.0 was used to construct a niche model based on vector surveillance data.

**Results:**

The spatial-temporal scan analysis revealed that the spatial distribution of malaria hotspots remained largely consistent from 2017 to 2023, with the exception of 2021(*P*<0.05). Persistent clusters were primarily located in Yaohai District, Luyang District, Baohe District, Feidong County, Changfeng County, Feixi County, and Shushan District. The ensemble model based on the probability committee average method demonstrated strong predictive performance, with an AUC of 0.943 and a TSS of 0.759. The results of the ensemble model showed that the potential suitable areas of *An. sinensis* were mainly distributed in the central and part of the southern regions. The distribution of *An. sinensis* was mainly affected by the annual vegetation index, the brightness of night lights, and the precipitation in the wetest month.

**Conclusions:**

The central region of Anhui Province is at the highest risk for malaria re-transmission. It is recommended to strengthen the surveillance of epidemic situations in the region, timely detection and standardized management of cases. Vector control measures should be taken in the epidemic spots during the mosquito vector activity season to interrupt the risk of re-transmission.

**Clinical trial number:**

Not applicable.

Malaria is a significant mosquito-borne disease caused by parasites of the genus *Plasmodium* [1–3]. It continues to be a significant global public health issue. Although substantial efforts have been directed toward malaria control, numerous countries continue to report indigenous cases. Furthermore, globalization has heightened the risk of imported malaria, posing a major public health threat and even leading to the re-transmission of malaria in some countries where it has been eliminated locally [4, 5]. China has long suffered from malaria epidemics, with widespread impacts that included serious long-term threats to public health and impediments to socioeconomic progres [6]. Situated within the Yangtze River Delta in southeastern China, Anhui Province constituted a historically significant endemic area for malaria. The province includes both temperate and subtropical climate zones [7, 8]. In 2006, the cases in Anhui Province accounted for more than half of the total number in China, and the incidence and number of cases ranked first in China [6, 9]. Through comprehensive control measures, Anhui Province has reported no locally transmitted malaria cases since 2014. By 2019, the province achieved sub-national malaria elimination certification. However, imported malaria cases persist. Additionally, *Anopheles sinensis*(*An. sinensis*), the vector for malaria, remains extensively distributed throughout Anhui Province, posing significant challenges to sustaining the achievements gained in malaria elimination [10].

This study investigates the spatiotemporal distribution of imported malaria and aims to forecast potential suitable habitats for *An. sinensis* in Anhui Province by utilizing spatial and temporal analyses alongside niche models. The findings visually represent the risk of malaria re-transmission, thus providing crucial guidance for preventing the re-transmission of malaria in the region.

## Methods

### Data collection

Epidemic data on imported malaria from January 1, 2017 to December 31, 2023 were from the infectious disease information reporting management system and parasitic disease information reporting management system. Demographic data for Anhui Province from 2017 to 2020 were collected via the Anhui Statistical Yearbook (http://tjj.ah.gov.cn/ssah/qwfbjd/rksj/index.html). County-level data for 2021–2023 were projected by the linear trend observed in the data from 2017 to 2020. The map of Anhui Province was sourced from the National Center for Basic Geographic Information (https://www.webmap.cn).

Based on existing research findings, data on key factors that may influence the distribution of *An. sinensis* were collected from several sources [11–13]. The main sources of the data including: Resource and Environmental Science Data Platform (https://www.resdc.cn), Worldclim (https://www.worldpop.org/) and Socioeconomic Data and Applications Center (SEDAC) (https://sedac.ciesin.columbia.edu/).

### Statistical analysis

#### Spatial autocorrelation analysis

The software of Arc Gis10.8 was used to spatial clustering analysis. The global autocorrelation was represented by Moran’s *I* index, while the local autocorrelation was indicated by the Getis-Ord Gi^*^ index. The Moran’s *I* values ranged from -1 to 1 [14, 15]. The larger the absolute value, the stronger the spatial autocorrelation [16]. The Gi^*^ index is also known as the Z-score. The larger the absolute value of Z, the clustering of high or low values [17].

#### Space-time scan analysis

The Space-Time Scan Analysis utilizes the Poisson model through the application of SaTScan 10.1.2. For this study, the scanning unit was set at the county/district level, with the scanning period covering from January 1, 2017, to December 31, 2023, using a yearly time unit. The maximum scanning spatial and temporal windows were set at 20%, and the focus of the scan was on high-value clusters. After completion of the scan, the log likelihood ratio (LLR) was calculated using the actual reported imported malaria cases, within and outside the scanning window, compared to the expected incidence. A statistically significant difference was considered when *P* < 0.05 [18–21].

#### Modeling

All data analysis was conducted in R.4.4.0. To enhance the robustness and generalizability of the final model and to mitigate the influence of overfitting and multicollinearity, variables with correlation coefficients exceeding 0.8 were excluded [22–26].

By analyzing the vector monitoring results, we identified a total of 98 locations where *An. sinensis* was present in the study area in 2023. To compensate for the lack of non-presence sites, we utilized the Biomod2 built-in function five times to generate pseudo-absence sites within the study area, maintaining a 1:1 ratio with the presence sites. Both the presence points and the pseudo-absence points were then used to construct the model [27].

The study employed a range of modeling algorithms available within the Biomod2 package to construct multiple models. These algorithms included Classification Tree Analysis (CTA), Flexible Discriminant Analysis (FDA), Generalized Boosting Model (GBM), Generalized Linear Model (GLM), Multiple Adaptive Regression Splines (MARS), Maximum Entropy (MaxEnt), Random Forest (RF), Surface Range Envelope (SRE), and eXtreme Gradient Boosting (XGBoost) Training. Area Under the Curve (AUC) and the true skill statistic (TSS) were used to evaluate the model performance. By setting a stricter criterion of 0.85, we aim to ensure that only individual models with sufficiently high performance are integrated [28]. This approach can establish a robust foundation for the final ensemble model.

In this study, 75% of the total point data was used for model construction, while the remaining 25% were reserved for model evaluation. The model construction process was repeated 10 times, resulting in a total of 450 models (9 algorithms * 10 repetitions * 5 runs per repetition = 450) [29]. Models with an AUC value greater than 0.85 from the individual models were selected. Based on these selected models, ensemble species distribution models were constructed using both probability-weighted averaging and voting averaging methods.

### Availability of data and materials

The dataset analyzed during the current study is available from the corresponding authors upon reasonable request.

## Results

### Spatial autocorrelation analysis

#### Global autocorrelation

From 2017 to 2023, the Moran’s *I* indices were all greater than 0, with annual values ranging from 0.044501 to 0.273769. Except for the year 2021^1^, the *P*-values for all other years were less than 0.05, indicating statistical significance (Table 1).

**Table 1:** Spatial global autocorrelation analysis of imported malaria cases in Anhui Province, 2017–2023

Year	*I*	*Z*	*P*
2017	0.198061	4.811048	<0.001
2018	0.234606	4.966756	<0.001
2019	0.200637	3.892974	<0.001
2020	0.216859	4.233150	<0.001
2021	0.044501	1.003618	0.315563
2022	0.140550	2.593878	0.009490
2023	0.273769	4.921334	<0.001

#### Hot Spot Analysis(local autocorrelation analysis)

**Fig. 1:**
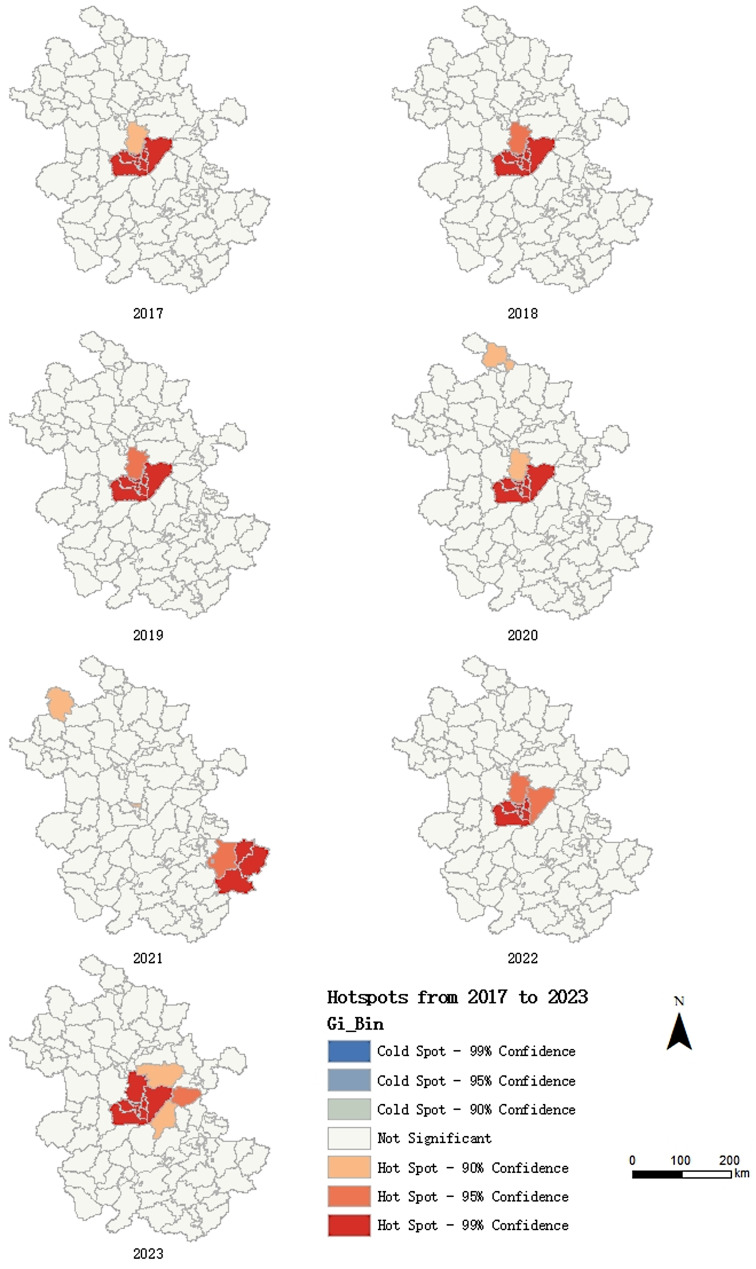
Hot Spot Analysis Map of Imported Malaria Cases at County Level in Anhui Province, 2017–2023

Through the Hot Spot Analysis of the distribution of imported malaria cases in Anhui Province from 2017 to 2023, it was found that the hot spot areas which mainly included Yaohai District, Luyang District, Baohe District, Feidong County, Changfeng County, Feixi County and Shushan District tended to be consistent from 2017 to 2023, with the exception of 2021 (Fig. 1).

### Space-time scan analysis

The spatial-temporal scan analysis of the incidence of imported malaria cases in Anhui Province from 2017 to 2023 showed that there were four levels of clustering areas. The first level of concentration area had a concentration time of 2018, covering 5 districts (counties), including Yaohai District, Luyang District, Baohe District, Feidong County, and Shushan District (*RR* = 12.37, *LLR* = 80.09, *P* = 0.001). The second level of concentration area had a concentration time of 2019, occurring in Tongguan District (*RR* = 22.60, *LLR* = 19.37, *P* = 0.001). The third level of concentration area had a concentration time of 2023, occurring in Yingjiang District (*RR* = 17.73, *LLR* = 9.63, *P* = 0.023). The fourth level of concentration area had a concentration time of 2021, occurring in Guangde District (*RR* = 8.15, *LLR* = 4.87, *P* = 0.778). (Table 2).

**Table 2:** Space-time scan analysis of malaria incidence in different county-level areas of Anhui Province from 2017 to 2023

Cluster	Duration(Year)	Cluster areas	Radius (km)	Cases	Expected	Relative Risk	Log likelihood ratio	*P*
1	2018	Yaohai District, Luyang District, Baohe District, Feidong County, and Shushan District	28.62	52	4.74	12.37	80.09	0.001
2	2019	Tongguan District	0	9	0.41	22.60	19.37	0.001
3	2023	Yingjiang District	0	5	0.29	17.73	9.63	0.023
4	2021	Guangde Distric	0	4	0.49	8.15	4.87	0.778

### Potential distribution of *An. sinensis* and analysis of model environmental variables

#### The variables for modeling

**Fig. 2:**
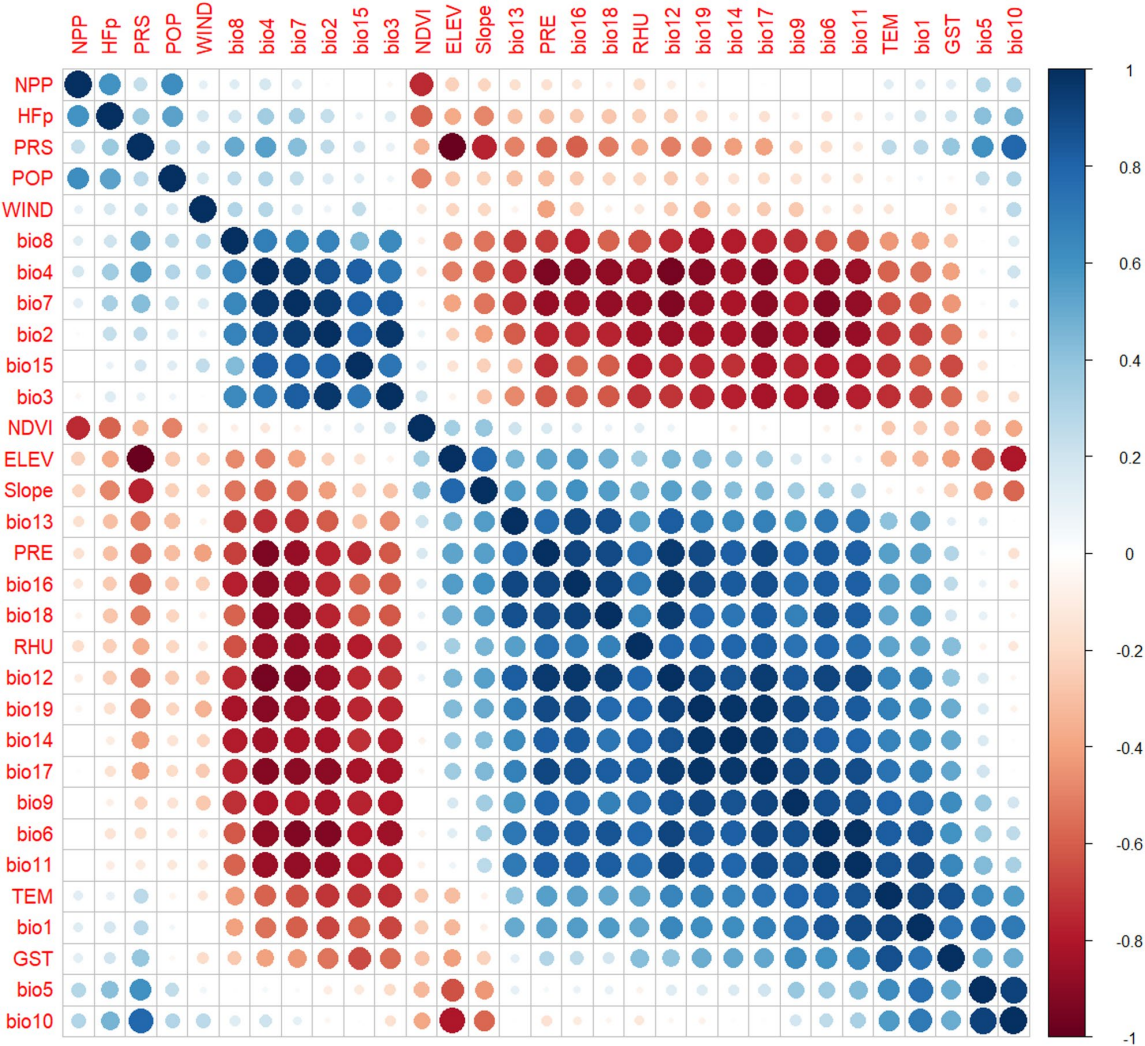
Pearson correlation analysis of variables

By calculating the correlation coefficient between two variables and excluding variables greater than 0.8, a total of 16 variables were finally included(Fig. 2; Table 3).

**Table 3:** Variables included in the model

Category	Variable name	Unit	Definition
Climate variables	bio3	-	Isothermality
	bio5	°C	Maximum temperature of warmest month
	bio8	°C	Mean temperature of wettest quarter
	bio11	°C	Mean temperature of coldest quarter
	bio13	mm	Precipitation of wettest month
	bio14	mm	Precipitation of driest month
	bio15	-	Precipitation seasonality
	GST	°C	Annual mean low temperature
	RHU	%z	Annual mean relative humidity
	WIND	m/s	Annual mean wind speed
Geographic variables	NDVI	-	Annual normalized difference vegetation index
	ELEV	m	Elevation
	Slope	°	Slope
Socio-economic variables	HFp	-	Human Footprint Index
	NPP	-	Night‑time lights
	POP	-	Spatial Distribution of Population

#### Model performance

**Fig. 3:**
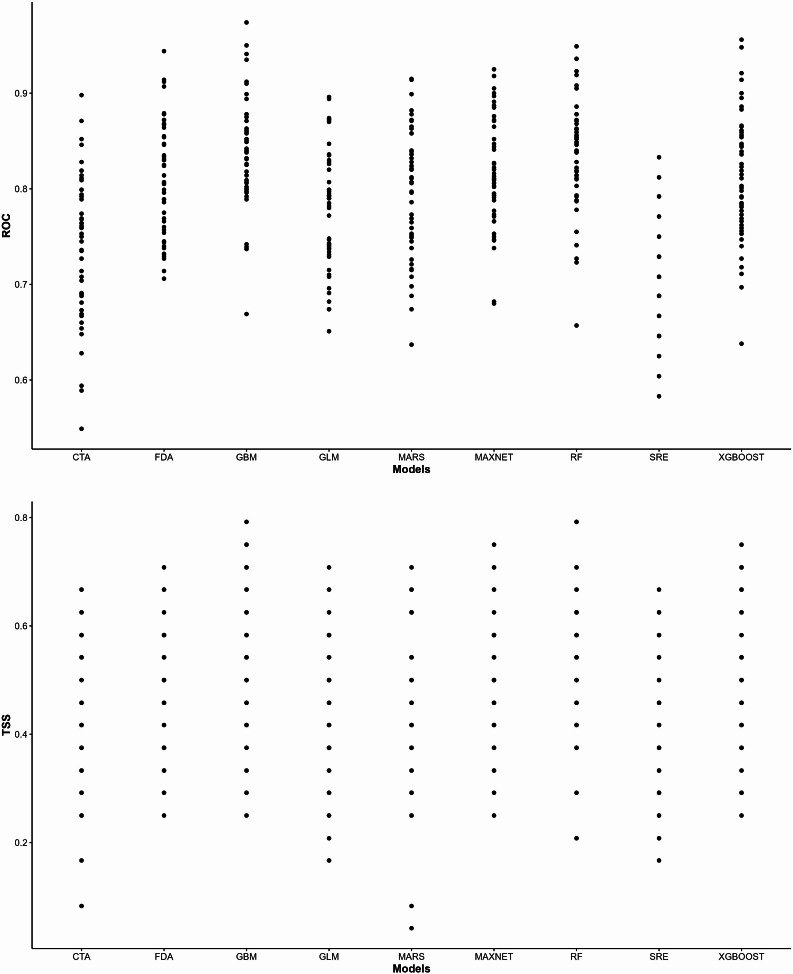
Values of ROC and TSS for a single model

This study used the AUC value and TSS value to evaluate the model performance of the results of both individual models and integrated models, as shown in Fig. 2. Among the 9 individual models, the GBM model had the highest model performance (AUC = 0.839 ± 0.056, TSS = 0.519 ± 0.123), followed by the RF model (AUC = 0.837 ± 0.058, TSS = 0.516 ± 0.124). The average AUC values were greater than 0.8 and the average TSS values were greater than 0.5 for all models. The SRE model had the lowest model performance (AUC = 0.701 ± 0.058, TSS = 0.401 ± 0.115) (Table 4; Fig. 3).

This study used Biomod2 to screen the single model in order to construct an integrated model to avoid the shortcomings of the single model and improve the model performance of prediction results, and reduce the bias caused by the single model [13]. The model performance of the ensemble models built using the probability weighted average (WM) and committee averaging (CA) methods is as follows: CA: AUC = 0.954, TSS = 0.759, WM: AUC = 0.943, TSS = 0.759. Overall, the ensemble model built using the CA method performs better, so the analysis of the results of the ensemble model in the following section will only discuss the model built using the CA method.

**Table 4:** Mean values of ROC and TSS for a single model

Model	AUC		TSS	
	Mean	SD	Mean	SD
CTA	0.740	0.075	0.430	0.136
FDA	0.814	0.060	0.473	0.117
GBM	0.839	0.056	0.519	0.123
GLM	0.779	0.062	0.424	0.130
MARS	0.793	0.067	0.447	0.140
MAXNET	0.819	0.058	0.498	0.112
RF	0.837	0.058	0.516	0.124
SRE	0.701	0.058	0.401	0.115
XGBOOST	0.812	0.066	0.493	0.126

#### Variable contribution and response curves

**Fig. 4:**
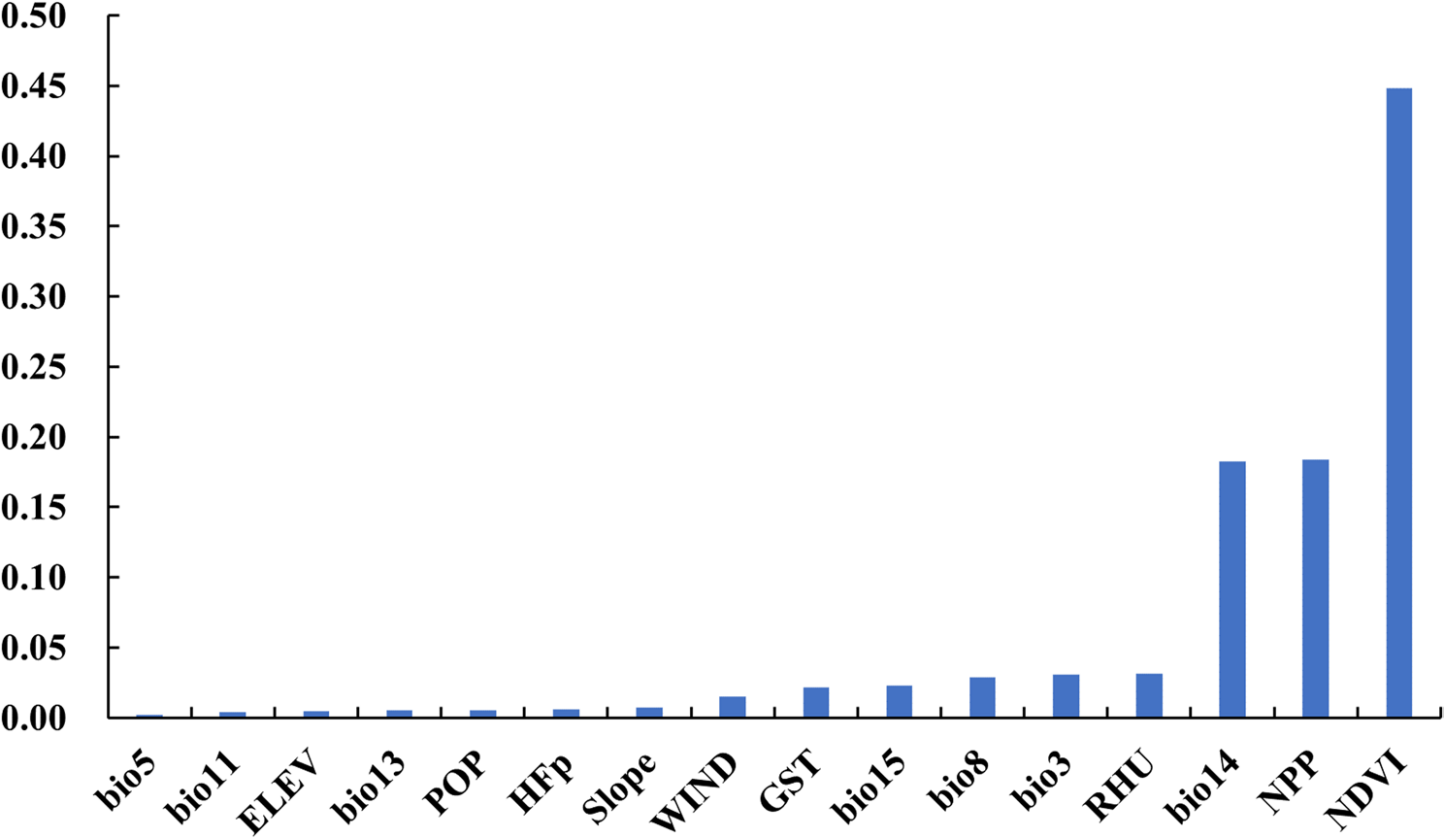
Relative contribution of environmental variables in ensemble model

In this study, sixteen variables were included to participate in the construction of the model, and the Biomod2 package was used to calculate the weight of each variable in the integrated model and normalize it. The results showed that the contribution of annual normalized difference vegetation index, night‑time lights, and precipitation of driest month were the highest, and the sum of these contributions accounted for the majority(Fig. 4).

**Fig. 5:**
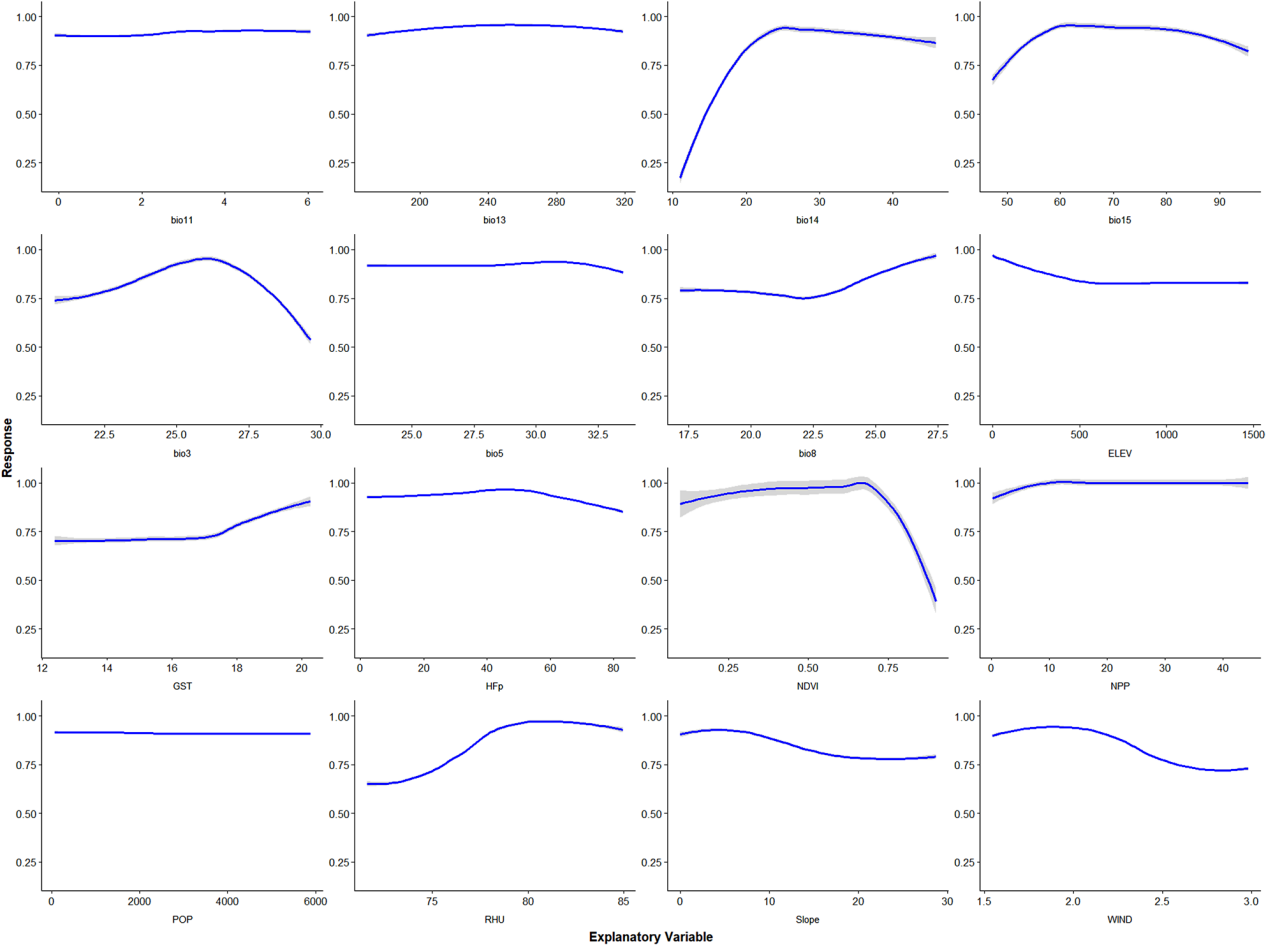
Response curves of environmental variables in integrated model (gray with 95%CI)

Further analysis of the environmental response curves predicted by the integrated model indicated distinct trends: the NDVI peaked at around 0.65 before declining, the driest month precipitation peaked at about 25 mm before declining, and the night-time light brightness remained relatively stable. (Fig. 5).

#### The suitable distribution area of *An. sinensis*

**Fig. 6:**
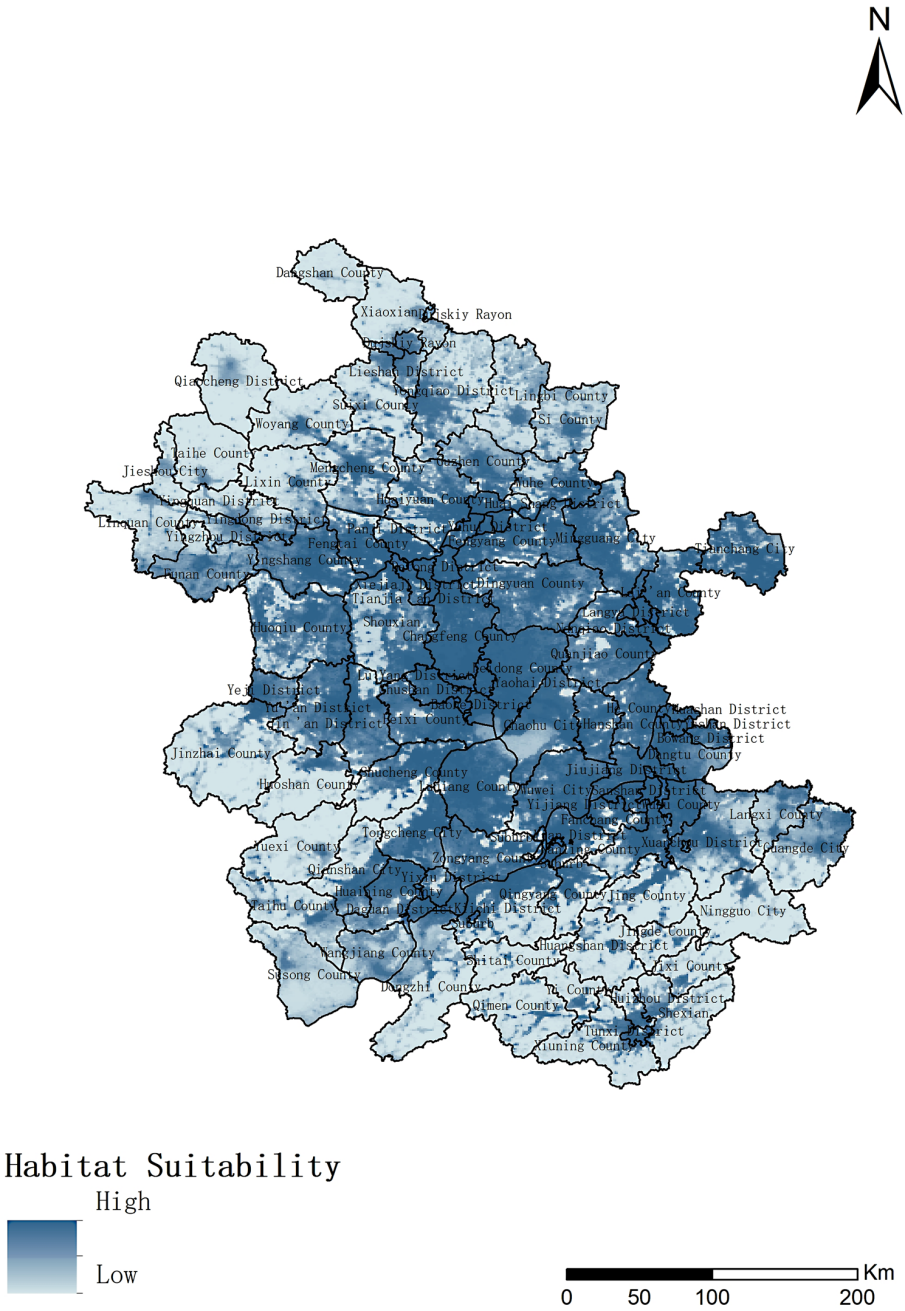
Fitting suitable distribution areas of *Anopheles sinensis* in Anhui Province

The prediction results of the ensemble model showed that the suitable distribution areas of *An. sinensis* were mainly distributed in the central region of Anhui Province, and the most suitable areas were Yaohai district, Luyang District, Baohe District, Feidong County, Shushan District, Changfeng County and Lujiang County. Compared with the central region of Anhui Province. The suitability of the southern, northern and southwestern regions was relatively low (Fig. 6).

## Discussion

Anhui Province has historically been a high-endemic area for malaria and a major labor-exporting region in China. In recent years, the province has frequently reported cases of imported malaria. Given that its ecological environment has remained largely unchanged and the population is still generally susceptible, conditions conducive to malaria transmission persist, posing a risk of re-transmission. This study visualizes the risk of malaria re-transmission in Anhui Province by identifying hotspot areas of imported malaria and predicting suitable distribution regions for *An. sinensis*.

The results of a global autocorrelation analysis indicated that the distribution of imported malaria cases in Anhui Province exhibited spatial clustering. However, an exception occurred in 2021 when practical factors, such as restrictions on movement during the initial phase of COVID-19, affected this clustering. The districts of Luyang, Yaohai, Shushan, Baohe, Feixi, Feidong, and Changfeng were identified as hotspots for imported malaria cases in Anhui Province. Space-time scan analysis further indicated that Yaohai, Luyang, Baohe, Feidong, and Shushan districts were the primary clustering areas. Consequently, imported malaria cases in Anhui Province were predominantly concentrated in Hefei City, particularly in the central region. This concentration is attributed to Hefei being the capital and economic center of the province, housing its most advanced medical resources. Patients with suspected symptoms or complex diagnostic cases often choose hospitals in Hefei for treatment. Additionally, Hefei’s developed transportation infrastructure facilitates international travel.

Anopheles is the main potential threat to malaria control and elimination, and the surveillance can better assist in the implementation of corresponding control measures [30]. In recent years, with the development of social economy, people’s living conditions and living environment have changed greatly. Changes in ecology and climate have certain impacts on the suitable distribution areas of media. Thus, in this study, we constructed a niche model based on the survellience data of *An. sinensis* in Anhui Province in 2023, using the data of climate, geographic environment and socioeconomic variables that might be affected by the monitoring results. The ensemble model fitting results showed that the main distribution areas of *An. sinensis* included the hot spots of imported malaria cases. In addition, the model results showed that among the environmental variables that form the integrated model, annual normalized difference vegetation index had the highest contribution, followed by Night‑time lights and Precipitation of driest month, indicating that vegetation coverage and growth activity, light and precipitation had a great impact on the distribution of *An. sinensis*, which were the same as the research results of Tong et al. [11] and Adeogun et al. [31].In addition, it is worth noting that the response curve results show that when the annual normalized difference vegetation index is high, there is a decline in suitability, which may be due to the lack of water conditions or other environmental conditions suitable for the growth and reproduction of mosquitoes despite the high vegetation density, so the high vegetation index is not suitable for their distribution [32, 33].

This study has two primary limitations. First, the risk of malaria transmission is highly dynamic and shaped by various environmental and socio-economic factors. The findings presented in this study reflect the transmission risk specific to Anhui Province during the study period and should be regularly updated based on ongoing surveillance data. Second, the scope of the study was limited to Anhui Province, which has unique geographical features, topography, and economic development levels that may not be representative of other regions. Consequently, extending the applicability of these results to other areas necessitates a careful assessment of local conditions and contextual factors.

## Conclusion

In conclusion, while local malaria cases have been eradicated in Anhui Province, the risk of re-transmission persists and exhibits regional heterogeneity. Therefore, focusing on imported malaria cases by providing tailored guidance and interventions that align with local conditions is essential. Since both imported malaria cases and vectors are chiefly concentrated in the central region of Anhui Province, enhanced intervention measures are warranted in this area. Key strategies should include improving the timely detection of malaria cases in medical institutions, reducing the misdiagnosis rate, and standardizing treatment protocols. Effective case management and increasing residents’ awareness of diagnosis and treatment are crucial [6, 34, 35]. Furthermore, vector monitoring should be strengthened to track critical indicators such as vector population and density. In the event of malaria cases, it is crucial to implement mosquito control measures, particularly during peak vector periods, to enhance vector control and minimize transmission risk [36–38].

## Data Availability

The dataset analyzed during the current study is available from the corresponding authors upon reasonable request.

## Footnotes

1 The discussion of the exception is presented in the Discussion section.

## Abbreviations

*An. sinensis*: *Anopheles sinensis*
LLR: Log likelihood ratio
RR: Relative risk
CTA: Classification Tree Analysis
FDA: Flexible Discriminant Analysis
GBM: Generalized Boosting Model
GLM: Generalized Linear Model
MARS: Multiple Adaptive Regression Splines
MaxEnt: Maximum Entropy
RF: Random Forest
SRE: Surface Range Envelope
XGBoost: eXtreme Gradient Boosting
AUC: Area Under the Receiver Operating Curve
TSS: True skill statistic
bio3: Isothermality
bio5: Maximum temperature of warmest month
bio8: Mean temperature of wettest quarter
bio11: Mean temperature of coldest quarter
bio13: Precipitation of wettest month
bio14: Precipitation of driest month
bio15: Precipitation seasonality
GST: Annual mean low temperature
RHU: Annual mean relative humidity
WIND: Annual mean wind speed
NDVI: Annual normalized difference vegetation index
ELEV: Elevation
HFp: Human Footprint Index
NPP: Night‑time lights
POP: Spatial Distribution of Population
WM: Weighted average
CA: Committee averaging
95%CI: 95% Confidence Interval
